# Recombination activating gene-2^null^ severe combined immunodeficient pigs and mice engraft human induced pluripotent stem cells differently

**DOI:** 10.18632/oncotarget.20626

**Published:** 2017-09-02

**Authors:** Yun-Jung Choi, EunSu Kim, Abu Musa Md Talimur Reza, Kwonho Hong, Hyuk Song, Chankyu Park, Seong-Keun Cho, Kiho Lee, Randall S. Prather, Jin-Hoi Kim

**Affiliations:** ^1^ Department of Stem Cell and Regenerative Biotechnology, Humanized Pig Research Center (SRC), Konkuk University, Seoul, Republic of Korea; ^2^ Department of Animal Science, Pusan National University, Miryang, Gyeongnam, Republic of Korea; ^3^ Department of Animal and Poultry Sciences, Virginia Tech, Blacksburg, VA, USA; ^4^ Division of Animal Science, University of Missouri-Columbia, Columbia, MO, USA

**Keywords:** severe combined immunodeficient, SCID, recombination activating gene-2, RAG-2, Talen, Immunology and Microbiology Section, Immune response, Immunity

## Abstract

This study comparatively investigated the transcriptional, physiological, and phenotypic differences of the immune disorder between severe combined immunodeficient (SCID) mouse and pig models. We discovered that the recombination activating gene-2 (*Rag-2*) SCID mice, but not *RAG-2* SCID pigs, showed intense, infrequent, and mild cluster of CD3^+^-, CD4^+^-, and CD8^+^ signals respectively, suggesting that distinct species-specific effects exist. Furthermore, the expression of six relevant genes (*NFATC1*, *CD79B*, *CD2*, *BLNK*, *FOXO1*, and *CD40)* was more downregulated than that in the *Rag-2* SCID mice, which provides a partial rationale for the death of T/B cells in the lymphoid organs of *RAG-2* SCID pigs but not in *Rag-2* SCID mice. Further, NK cell maturation-related gene expression was significantly lower in *RAG-2* SCID pigs than in *Rag-2* SCID mice. Consistently, the *RAG-2* SCID pigs, but not *Rag-2* SCID mice, developed human induced pluripotent stem cell-derived teratomas that were the same as those of *perforin*/*Rag-2* SCID mice. Therefore, these unexpected findings indicate the superiority of *RAG-2* SCID pigs over *Rag-2* SCID mice as a suitable model for investigating human diseases.

## INTRODUCTION

Severe combined immunodeficiency (SCID) is a genetic disorder of the functions of major lymphocytes such as B- and T-cell impairment [1, 2]. The SCID mouse model has been used to study human diseases; however, it has some limitations in mimicking human disease pathologies, such as their different body size, longevity, physiology, metabolism, and xenobiotic receptors [3. 4, 5, 6]**.** Our group and others previously reported that interleukin 2-receptor subunit gamma (*IL2RG*)-mutated pigs possess numerous cluster of differentiation 3^+^ (CD3^+^) cells and a moderate amount of B-cells although they did not have T and natural killer (NK) cells in their system [7, 8, 9]. Another study reported that *Il2rg*^*−/Y*^ hemizygous pigs do not reject allografts and allow the limited engraftment of xenografts [10], indicating that *Il2rg*^*−/Y*^ hemizygous pigs are much less efficient in accepting engrafts of human cells.

To overcome the limitations of the *IL2RG*-mutated pig model, we and other groups developed SCID pigs deficient in two alleles of the recombination activating gene 2 (*RAG-2*, biallelic knockout SCID pigs; hereafter referred to as *RAG-2* bKO pigs) to fill the gap in knowledge between mice and humans [11, 12, 13]. In this study, the *RAG-2* bKO pigs, similar to perforin (*Pfp*)/*Rag-2* double knockout (dKO) mice (T^-^/B^-^/NK^-^ cells), formed human inducible pluripotent stem (hIPS) cell-derived teratomas, whereas the *Rag-2* KO SCID mice (T^-^/B^-^/NK^+^ cells; hereafter referred to as *Rag-2* KO mice) did not. This was unexpected because unlike *Rag-2* KO mice, *RAG-2* bKO pigs can be used as human disease models. Thus, why modification of the *RAG-2* gene alone is sufficient to allow engraftment of hIPS cells in these pigs remains to be explained. To elucidate the molecular mechanism involved in SCID pig lymphoid organ functions, we compared *RAG-2* bKO pigs with *Rag-2* KO or *Pfp*/*Rag-2* dKO mice.

## RESULTS AND DISCUSSION

**Figure 1:**
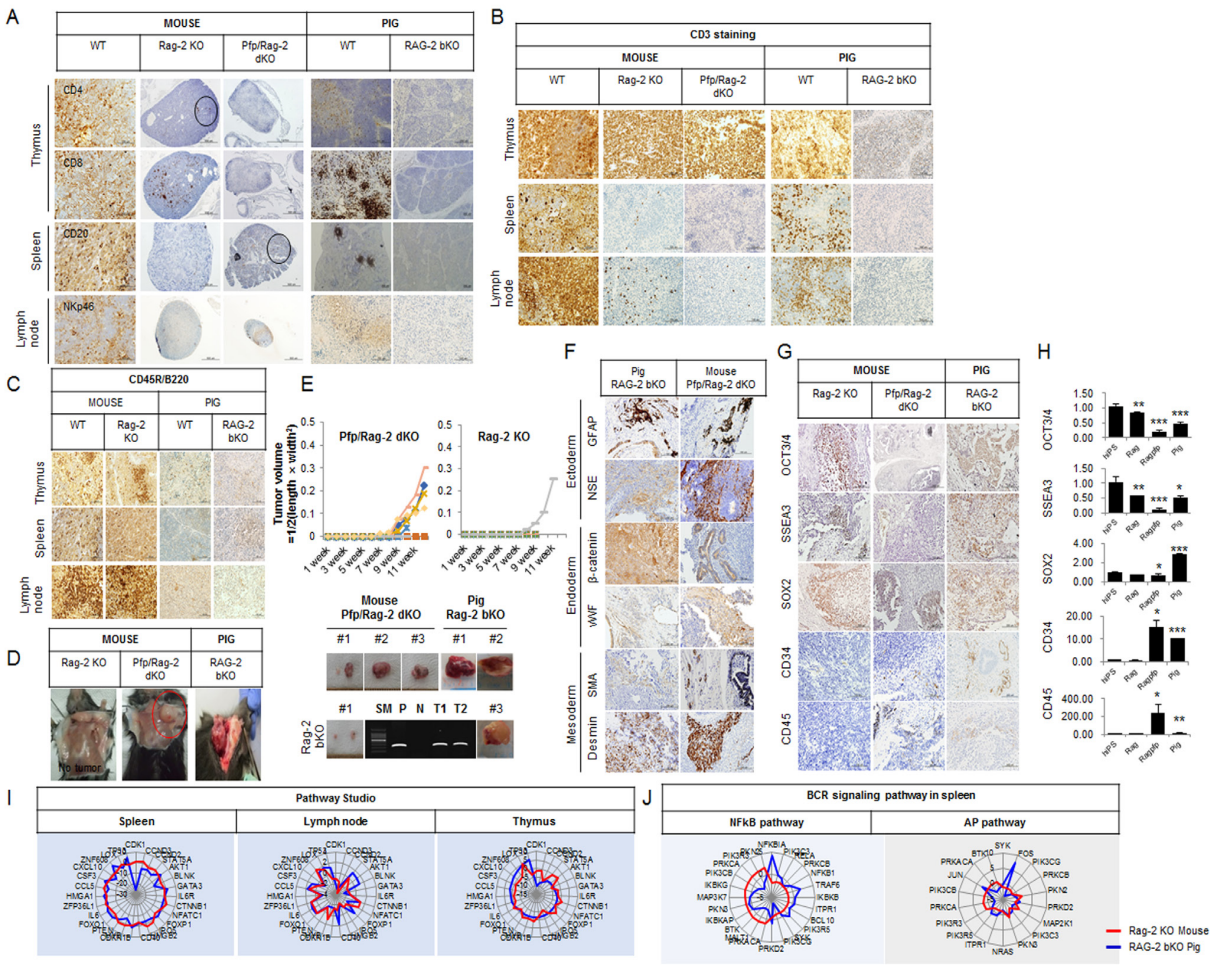
Xenogeneic hIPS cell fate transplanted into *Rag-2* KO mice, *Pfp/Rag-2* dKO mice, and *RAG-2* bKO pigs **A.** Immunohistochemical analysis of T-, B-, and NK cell biomarkers in the thymus, spleen, and lymph node, respectively. **B.** and **C.** Immunohistochemical analysis of CD3^+^ and CD45R/B220 expression. **D.** Teratoma formation in *Rag-2* KO mice, *Pfp*/*Rag-2* dKO mice, and *RAG-2* bKO pigs. **E.** Comparison of tumor growth curves of *Pfp*/*Rag-2* dKO and *Rag-2* KO mice. Bottom panel shows teratomas from *Pfp*/*Rag-2* dKO mice, *Rag-2* KO mice, and *RAG-2* bKO pigs, respectively. To identify the source of teratomas, the human-specific mitochondrial mitofusin 1 (*MFN1*) gene was amplified using PCR. SM, P, N, T1, and T2 indicate size marker, positive control, negative control, teratoma sample 1, and teratoma sample 2 recovered from *RAG-2* bKO pigs, respectively. **F.** Three germ-layer analysis in *Pfp*/*Rag-2* dKO and *Rag-2* KO mouse-derived teratomas. **G.** Immunohistochemical and **H.** RT-qPCR analyses of pluripotency-related marker gene expression levels in *Rag-2 KO* mice-, *Pfp/Rag-2* dKO mice-, and *RAG-2* bKO pig-derived teratomas. hIPS cells were used as positive control. hIPS, Rag, Rag/Pfp, and pig indicate hIPS cells, *Rag-2* KO mice-, *Pfp/Rag-2* dKO mice-, and *RAG-2* bKO pigs-derived teratomas, respectively. **I.** Pathway Studio analysis of mRNA expression patterns illustrated using radar charts. Red and blue lines represent *Rag-2* KO mouse and *RAG-2* bKO pig, respectively.**J.** Radar chart analyses of BCR signaling pathway-related gene expressions in *RAG-2* bKO pig- and *Rag-2* KO mouse-derived spleens.

Previously, we reported that most *RAG-2* bKO pigs are athymic [11]. In this study, we found that the size of the spleen of *RAG-2* bKO pigs was smaller than that of the age-matched control pigs, whereas the difference was not obvious in the *Rag-2* KO and wild-type (WT) mice (Supplementary Figure 1A and 1B). Of note, the *RAG-2* bKO pigs presented a T^-^B^-^NK^low^ SCID phenotype with a remarkably reduced level of NK cells (Supplementary Figure 1B). However, not many CD4^+^, a few CD8^+^, and numerous NKp46^+^ cells were detected in the *Rag-2* KO mice (Figure 1A), while a significant number of CD3^+^ T cells were present in the thymus, and a few were also detected in the lymph node (Figure 1B). However, these CD3^+^ and CD8^+^ cells were not found in the *RAG-2* bKO pigs. We next examined CD20^+^ and B220^+^ cells in both *Rag-2* mutated mice and pigs. As shown in Figure 1C, the spleens of WT and *Rag-2* KO mice, unlike those of the *RAG-2* bKO pigs, showed numerous CD20^+^ and B220^+^ cells. Thus, our results indicate that *Rag-2* KO mice developed an immune reactivity that is known as SCID leakiness.

As shown in Figure 1D and 1E, most of the *Rag-2* KO mice failed to form teratomas from engrafted hIPS cells, except for one mouse that formed a rudimentary teratoma structure. However, the *Pfp*/*Rag-2* dKO mice and *RAG-2* bKO pigs consistently supported the growth of teratoma, indicating differences in their capacities to support the growth of engrafted hIPS cells. The histological composition of teratomas from these animals was evaluated by identifying crucial biomarkers: glial fibrillary acidic protein (GFAP) and neuron-specific enolase (NSE) for the ectoderm, β-catenin and von Willebrand factor (vWF) for the endoderm, and smooth muscle actin (SMA) and desmin for the mesoderm. Teratomas derived from *Pfp*/*Rag-2* dKO mice and *RAG-2* bKO pigs contained several cells derived from these three germ layers (Figure 1F), whereas this was not detected in *Rag-2* KO mouse-derived teratoma-like structures. The results of the reverse transcription quantitative polymerase chain reaction (RT-qPCR) analysis were slightly different to those of the immunohistochemical analysis (Figure 1H). Specifically, the transcript levels of octamer-binding transcription factor 3/4 (*OCT3/4*), stage-specific embryonic antigen-3 (*SSEA3*), and sex determining region Y-box 2 (*SOX2*) in the *RAG-2* bKO pig-derived teratomas were higher than those in the *Pfp*/*Rag-2* dKO mouse teratomas were. Of note, a lower frequency of OCT3/4^+^ and SSEA3^+^ and higher CD34^+^ and CD45^+^ signals were detected in teratomas from *RAG-2* bKO pigs than in those from *Rag-2* KO or *Pfp*/*Rag-2* dKO mice (Figure 1G).

To identify V(D)J rearrangements in the T-cell receptor (TCR)-β and B-cell receptor (BCR) loci, we performed PCR. PCR products from the lymph node, spleen, and thymus of *RAG-2* bKO pigs showed a single band as a positive control, whereas the WT and *RAG-2* monoallelic knockout (mKO) pigs showed additional rearranged bands, indicating that the lymphoid organs of *RAG-2* bKO pigs lacked V(D)J rearrangement in the TCR-β locus (Supplementary Figure 1C). Next, the immunoglobulin H (IgH) locus of the BCR rearrangement was selected. Rearrangement of IgH was undetectable in *RAG-2* bKO pig, whereas the WT and *RAG-2* mKO pigs showed a rearranged band (Supplementary Figure 1D). Consistent with a previous finding (Huang et al., 2014), our results indicate that *RAG-2* bKO pigs did not exhibit recombination of the TCR-β and BCR loci and that the lack of mature T/B cells in the lymphoid organs of *RAG-2* bKO pigs might be caused by the inhibition of antigen receptor gene expression.

To identify the differential underlying mechanisms in both the *RAG-2* SCID pigs and mice, we performed microarray and RNA-sequence analyses (Supplementary Figure 1E∼1H). As shown in Supplementary Figure 1G, gene ontology (GO) analysis of the lymphoid organs of *RGA-2* SCID pigs and mice showed some common pathways including antigen processing and presentation, primary immunodeficiency, and T-cell receptor signaling pathways. Overall, however, the expression levels of genes involved in T-cell, lymphocyte, mononuclear leukocyte, leukocyte, and blood cell formation were significantly lower in the *RAG-2* bKO pigs than they were in the *Rag-2* KO mice (Supplementary Figure 1H and 1I). Although the studio pathways analysis showed similarities between the *RAG-2* bKO pigs and mice (Figure 1I), *AP-1* genes (Jun and Fos) expression, which is controlled by BCR signaling, was more greatly increased in *RAG-2* bKO pigs than it was in *Rag-2* KO mice (Figure 1J).

**Figure 2:**
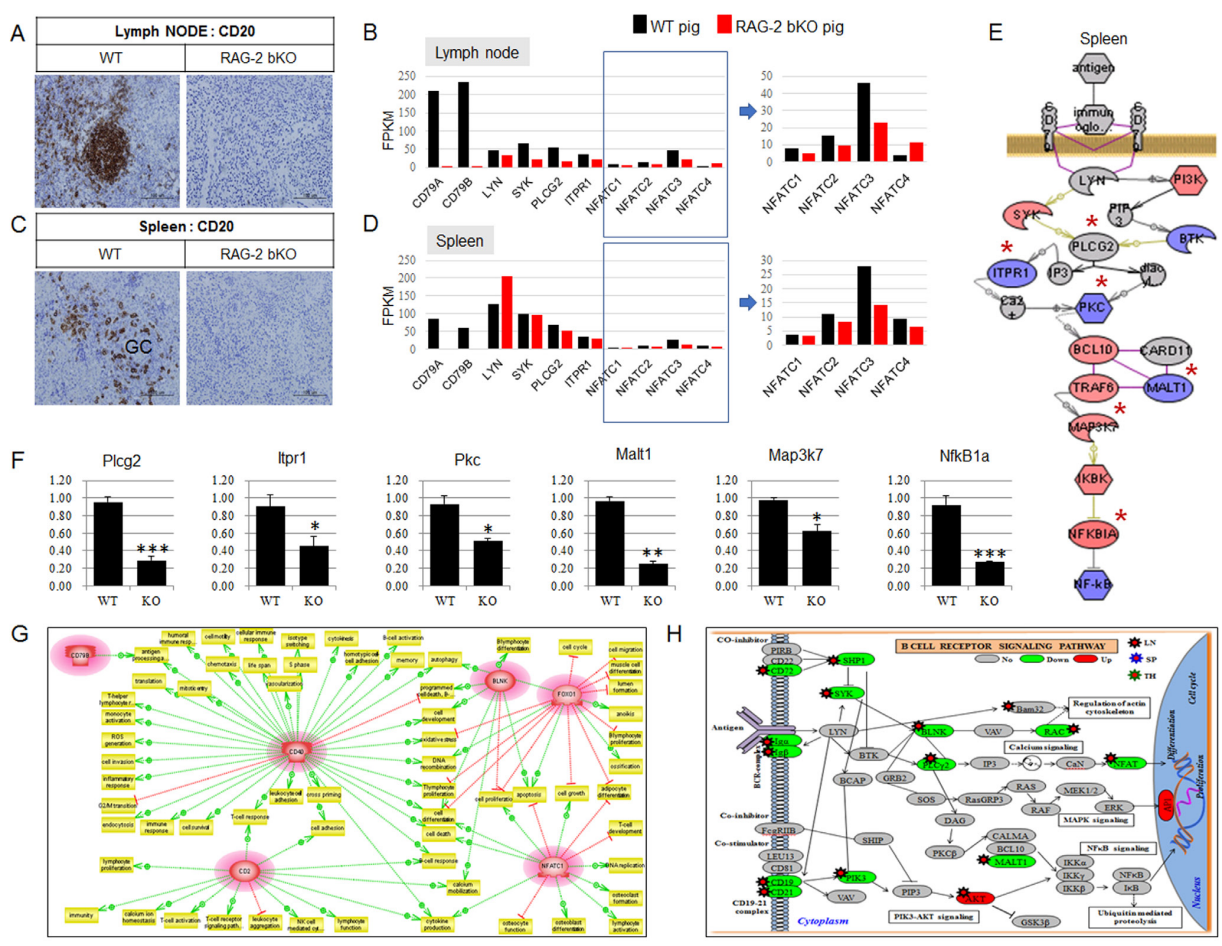
BCR-dependent/NFATCs signaling pathways analysis in lymph node and spleen of WT and *RAG-2* bKO pigs using microarray or RNA-sequence analysis **A.** and **C.** Comparison of CD 20 expression in lymph node and spleen of WT and *RAG-2* bKO pigs. **B.** and **D.** Comparison of BCR signaling-related gene expression between WT and *RAG-2* bKO pigs using RNA-sequence (RNA-Seq) analysis. Of note, key transcription factors, nuclear factor of activated T-cells (NFATCs), in *RAG-2* bKO pigs were significantly decreased compared to those of WT pigs. NFATCs in quadrangle are enlarged. **E.** BCR signaling analysis using microarray. Red, blue, and gray colors indicated increased, decreased, and not altered expression, respectively. **F.** Reverse transcription-polymerase chain reaction (RT-PCR) analysis. Six genes were selected based on signaling of (E*). Although *Map3k7* and *NfkB1a* gene expression in microarray analysis are significantly increased (red color), RT-PCR analysis showed that these gene expressions were significantly decreased. **G.** Six genes significantly dysmod in microarray analysis and their subnetwork analysis in *RAG-2* bKO pig lymphoid organs. These genes are shown by their gene symbols and significantly downregulated compared to WT pig or *Rag-2* KO mouse. Positive (green color) and negative (red color) regulation is indicated by dotted arrowed lines, respectively. **H.** BCR signaling pathway analysis in WT and *RAG-2* bKO pigs. Up/downregulated transcripts are depicted in red/green in this illustration. Grey color indicates not altered or detected gene expression between WT and *RAG-2* bKO pigs.

As shown in Figure 2A and 2C, CD20 expression in the lymph node and spleen of *RAG-2* bKO pigs was not detected, whereas the organs of WT pigs strongly expressed CD20. RNA-sequence analysis of the lymph nodes and spleens of *RAG-2* bKO pigs also demonstrated that BCR signaling-mediated nuclear factor of activated T-cell (*NFATC*) family 1, 2, and 3 gene expression levels were significantly downregulated (Figure 2B and 2D). Although microarray analysis showed slightly uneven gene expression profiles (Figure 2E), the RT-qPCR analysis showed that the expression levels of all *BCR*/*NFκB* signaling-related genes examined in the spleen of *RAG-2* bKO pigs were significantly decreased compared to those of WT pigs (Figure 2F). In this study, we identified six genes [*NFATC1*, *CD79B*, *CD2*, B-cell linker protein (*BLNK*), Forkhead box transcription factor class O1 (*FOXO1*), and *CD40*] that were commonly and greatly dysregulated in the lymphoid organs of *RAG-2* bKO pigs but not *Rag-2* KO mice. An analysis using the Pathway Studio network prediction program revealed an association of the six genes with lymphoid development (Figure 2G), indicating that any mechanism that contributes to reducing *NFATC* and *NFκB* gene expression levels in *RAG-2* bKO pigs, but not *Rag-2* KO mice, may decrease the risk for SCID leakiness. In addition, we detected more dysregulated gene expression levels (*CD19*, *CD21*, *CD72, BLNK*, *Igα*, *μH, EBF1, and PAX5*), which may be blocked from the pro-B to pre-B transition, in RAG-2 bKO pigs than we did in *Rag-2* KO mice (Figure 2H). Of note, since AP upstream gene expression levels such as those of mitogen-activated protein kinases (*MAPKs*), *p38*, extracellular signal-regulated kinase *ERK* (1/2) and c-Jun N-terminal kinase (*JNK*) were not affected, the reason for the observed upregulation of the *AP-1* genes (Fos and Jun) is not clear (Figure 2H). Overall, our findings provide a plausible explanation why few B220^+^ cells were detected in the lymphoid organs of *RAG-2* bKO pigs unlike those of *Rag-2* KO mice with ample B220^+^ cells.

**Figure 3:**
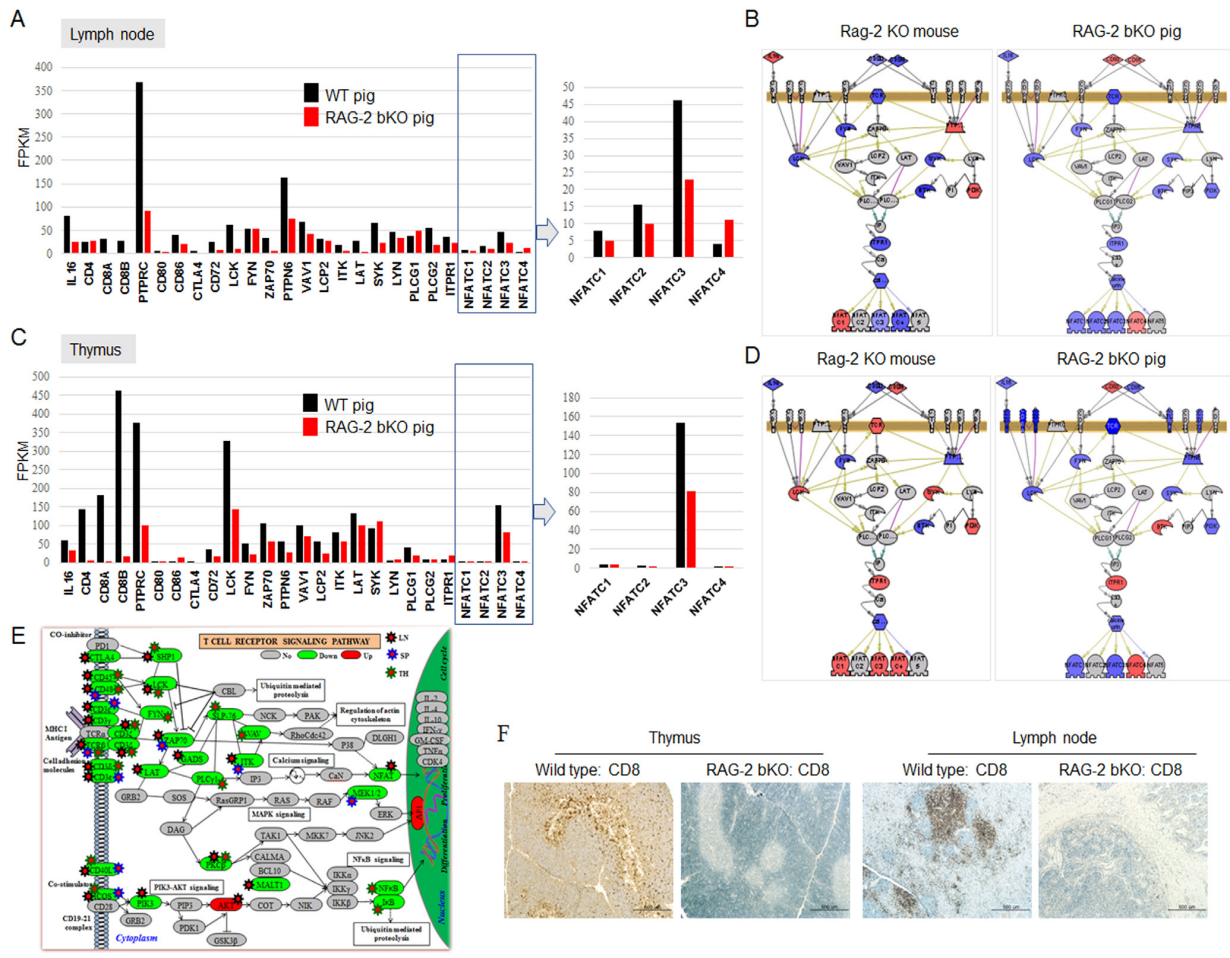
TCR-dependent/NFATCs signaling pathways analysis in lymph node and thymus of WT and *RAG-2* bKO pigs using microarray or RNA-sequence analysis **A.** and **C.** TCR/NFATC signaling pathway-related gene expression profiles in lymph node **A.** and thymus **C.** of WT and *RAG-2* bKO pigs using RNA-sequencing. **B.** and **D.** Different expression of *TCR*/*NFATCs* signaling-related genes in lymph node **B.** and thymus **D.** between *Rag-2* KO mice/pigs (left/right) and WT mice/pigs using microarray analysis. Red, blue, and gray indicate upregulation, downregulation, and not detected/altered expression, respectively. **E.** TCR signaling pathway gene expressions analysis using RNA-sequence. Red, green, and gray indicate upregulation, downregulation, and not detected/altered expression, respectively. **F.** CD8 expression pattern analysis in thymus and lymph nodes of WT and *RAG-2* bKO pigs.

Supplementary Figure 2A-2C shows the top 15, 8, and 4 enriched functional annotations that were significantly downregulated in the lymphoid organs of *RAG-2* bKO pigs. Supplementary Table 1 shows 15 signaling pathway molecules that were more differentially expressed in *RAG-2* bKO pigs than they were in *Rag-2* KO mice. Among them, we focused on *TCR*/*NFATC* gene expression levels, which not only regulate activation but also are involved in the control of thymocyte development, T-cell differentiation, and self-tolerance [14]. As shown in Figure 3A, *TCR* signaling and *NFATC* family 1, 2, and 3 gene expression levels in the lymph nodes of *RAG-2* bKO pigs were significantly downregulated, whereas the *NFATC4* gene, which is not expressed in T cells [15], showed a slight increase compared to of the WT pigs. This observation indicates that low levels of *NFATCs* in the nucleus was not sufficient to compensate for the deficiency of severely impaired multiple cytokine production due to the reduced binding of NFATCs to cytokine promoter elements. In the thymus, *NFATC1*, *2*, and *3* expression levels in *RAG-2* bKO pigs were significantly decreased or not altered (Figure 3C), whereas *NFATC1*, *3*, and *4* expression levels in *Rag-2 KO* mice were significantly increased (Figure 3B and 3D). This observation indicated that signal networks are conserved between species, but the details differ considerably. In addition, *TCRβ*, *CD48*, *CD45*, cytotoxic T-lymphocyte-associated protein (*CTLA*), inducible T-cell co-stimulator (*ICOS*), *CD40L* as well as *Artemis, CD3ζ*, *CD3ε,* and *CD3δ* gene expression levels, which are critical factors for pro-T to pre-T transition, in *RAG-2* bKO pigs were greatly dysregulated (Figure 3E). Consistent with these results, CD8^+^ cells were not detected in the lymph nodes and thymus in *RAG-2* bKO pigs (Figure 3F). Considering that *NFATC1 and NFATC4*, which are more highly expressed in *Rag-2* KO mice than in *RAG-2* bKO pigs, were found to play a role in the proliferative expansion of immature thymocytes [16] and positive selection [17], our results explain why few CD8^+^ (CD4^+^) cells were only detected in the *Rag-2* KO mice.

As shown in Supplementary Figure 2D, a higher level of genes involved in the cell cycle [Kinesin family member 11 (*Kif11*), Disks large-associated protein 5 (*Dlgap5*), DBF4 zinc finger (*Dbf4*), Targeting protein for Xklp2 (*Tpx2*), Aurora kinase A (*Aurka*), Centromere protein H (*Cenph*), RAD51 recombinase (*Rad51*), Cyclin B1 (*Ccnb1*), Transcription factor Dp-2 (*Tfdp2*), and Myosin heavy chain 10 (*Myh10*)] were detected in the spleens of *Rag-2* KO mice than in those of *RAG-2* bKO pigs. Similarly, the levels of genes involved in the regulation of cell death, apoptosis, the cell cycle process, lymphoid development, and response to wound healing showed remarkable differences between the *Rag-2* KO mice and *RAG-2* bKO pigs. These observations suggest that the absence of early/mature T cells in *RAG-2* bKO pigs may be caused by balance disruption of the cell cycle process, regulation of cell death, and apoptosis. Thus, these analyses partially explain why *RAG-2* bKO pigs had smaller spleens than the WT pigs did, whereas those of *Rag-2* KO and WT mice were similar in size.

**Figure 4:**
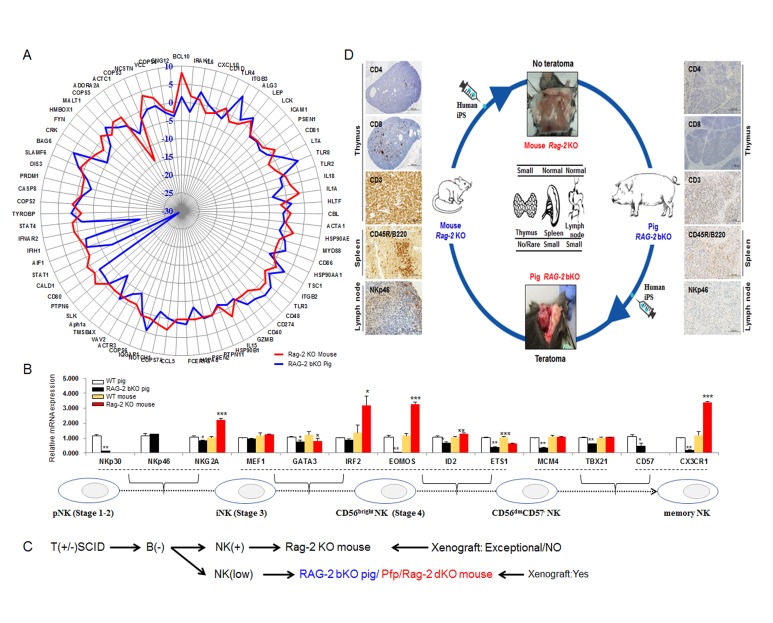
Different expression profiles of NK cell maturation-related genes between *Rag-2* KO mice and *RAG-2* bKO pigs **A.** Profile analysis of proliferation-related genes in NK cells of *Rag-2* KO mouse and *RAG-2* bKO pig-derived lymph nodes. Upregulated and downregulated gene expressions in *RAG-2* bKO pig lymph nodes compared with those of *Rag-2* KO mice. Blue and red lines indicate *RAG-2* bKO pig and *Rag-2* KO mouse, respectively. **B.** RT-qPCR analysis of NK cell maturation-related gene expression. Bottom panel indicates each stage of NK cell maturation. **C.** Flow diagram of the major events of xenograft rejection in the *Rag-2* KO mouse and *RAG-2* bKO pig. Of note, teratoma formation in *RAG-2* bKO pigs was comparable to that in *Pfp*/*Rag-2* dKO mice, but not *Rag-2* KO mice. **D.** Graphic conclusion between *Rag-2* KO mice and *RAG-2* bKO pigs. In *Rag-2* KO mice, numerous CD3^+^ and CD45R (B220), and few CD8^+^, NK46, and CD^+^4 cells were detected, whereas *RAG-2* bKO pigs did not show this expression. Further, *RAG-2* bKO pigs but not *Rag-2* KO mice formed hIPS cell-derived teratoma.

Finally, we compared the expression profiles of genes associated with NK cell maturation between WT and *Rag-2* KO mice or WT and *RAG-2* bKO pigs. NK cell maturation-related gene expression levels (Figure 4A) in the *RAG-2* bKO pigs were generally different compared with those in *Rag-2* KO mice, indicating a dysregulation of NK cell numbers. Of note, genes involved in the conversion of precursor NK (pNK) to immature NK cells (iNK; *NKp30*, *NKp46*, and *NKG2A*) or iNK to CD56^bright^ NK [GATA binding protein 3 (*GATA3*), Eomesodermin (*EOMOS*), Inhibitor of DNA binding 2 (*ID2*), ETS proto-oncogene 1 (*ETS1*), Minichromosome maintenance complex component 4 (*MCM4*), T-box 21 (*TBX21*)] and memory NK cells [C-X3-C motif chemokine receptor 1 (*CX3CR1*)] showed significantly lower expression levels in *RAG-2* bKO pigs than in WT pigs, However, the transcript levels of these genes were considerably higher in *Rag-2* KO mice than they were in WT mice or *RAG-2* bKO pigs (Figure 4B). Further, Adenosine deaminase (*ADA*)*, G*amma chain (*γc*)*,* and *IL7Rα* gene expression levels, which are essential factors for pNK cell development, were significantly dysregulated in *RAG-2* bKO pigs (Supplementary Figure 2E). Thus, our results indicate that the presence of a large number of NK cells in *Rag-2* KO mice, unlike that in *RAG-2* bKO pigs, could be the main hurdle for teratoma formation and subsequent hIPS cell differentiation. The schematic diagram in Figure 4C illustrates the differences in the xenografting potential of *Rag-2* KO mice and *RAG-2* bKO pigs. In particular, it shows that the *RAG-2* bKO pig, which has a similar phenotype to that of the *Pfp*/*Rag-2* dKO mouse, may be a more effective model than the *Rag-2* KO mouse.

## CONCLUSIONS

Although the benefits of SCID mice with human patient-derived cells are undeniable, there are some limitations to their use for long-term follow-up of certain disease states. On the contrary, SCID pigs with a longer lifespan may be a useful model for studying phenotypes associated with late-onset diseases or complex disease phenotypes, such as Alzheimer’s disease [18]. In this study, we performed a comparison of the expression profiles in lymphoid organs by microarray and RNA-Seq analysis, and found the similarities and differences in the transcriptome, protein levels, and tissue structure.

To the best of our knowledge, this is the first study comparing the expression profiles of lymphoid organs using microarray and RNA-sequence analyses as well as teratoma efficiency and histological composition between *RAG-2* SCID pigs and mice. In conclusion, our previously reported IL2RG mutated pigs contained CD3^+^ positive cells and moderate B cells although they did not have T and NK cells in their system [7]. In addition, another study reported that IL2RG^-/Y^ mutated pigs failed to form teratomas from engrafted human iPS cells [10]. In this study, RAG2^-/-^ pigs represented T^-^B^-^NK^+^ SCID phenotype with a remarkably reduced level of NK and CD3^+^ cells compared to Rag2^-/-^ mice. These RAG2 SCID pigs successfully formed human iPS-derived teratoma whereas Rag2^-/-^ mice or IL2RG^-/Y^ mutated pigs failed to form teratoma. Interestingly, Rag2/Pfp dKO mice successfully formed teratoma from the identical source of human iPS cells. This study depicted a clear phenotypic difference between RAG2^-/-^ SCID pig and mouse or between Rag2 and Rag2/Pfp KO mice in relation to the presence of CD3^+^ T and B cells or CD8^+^ T and NK cells, respectively. Overall, our study indicates that the absence of CD8^+^ T and NK cells in mice and CD3^+^ T/B cells in pigs is a critical component in the formation of teratoma from hIPS cells (Figure 4D). Thus, this newly developed *RAG-2* bKO SCID pig model would be a valuable resource in various biomedical fields such as stem cell, cancer, and translational research.

## MATERIALS AND METHODS

### Animal source and care

All animal experimental protocols were approved by the Institutional Animal Care and Use Committee of Konkuk University (IACUC approval numbers: KU12044 for mice and KU15085 for pigs). Recombination activating gene-2 (*Rag-2*) knockout (KO) and perforin (*Pfp*)/*Rag-2* double KO (Dko, B6.129S6-*Rag2*^*tm1Fwa*^*Prf1*^*tm1Clrk*^ N12) mice were obtained from Taconic Farms Inc., (http://www.Taconic.com). The *RAG-2* bKO pigs were described in our previous report [11].

### Teratoma formation and analysis

Teratoma formation was analyzed as described previously [11, 19]. Cultured human inducible pluripotent stem (hIPS) cells were detached using dispase (StemCell, Cat. no. 07923) and scraping. After centrifugation (200 × *g*, 5 min), the cell pellet was resuspended in 0.2 mL mTeSR1 medium (StemCell, Cat. no. 85821, 85852) and mixed with 25% Matrigel solution (BD, Cat. no. 354277). hIPS cells were subcutaneously injected into the both axilla regions of *Rag-2* KO and *Pfp/Rag-2* dKO mice, and one each in the ear and lateral flank of the *RAG-2* bKO pigs. Approximately four million cells were used for *Rag-2* KO and *Pfp/Rag-2* dKO mice, whereas ten million cells were injected into the *RAG-2* bKO pigs. The transplanted animals were observed routinely once weekly, and tumor growth was measured using a caliper. For teratoma analysis, each teratoma was surgically removed from the euthanized animals and fixed in 10% buffered formalin. After serial washing, each teratoma was embedded in paraffin, sectioned, and stained with specific antibodies. For co-staining, hematoxylin and eosin (H&E) was used. All photomicrographs were acquired using an Olympus microscope equipped with a DP70 high-resolution digital microscope camera.

### Immunohistochemical analysis

Immunohistochemical analysis of the thymus, spleen, and lymph node was performed as described previously [11]. Tissues were fixed in neutral buffer containing 10% formalin, and slides were prepared for the analysis. The endogenous peroxidase activity of the samples was blocked with 3% hydrogen peroxidase, followed by pretreatment with Borg Decloaker (Biocare, Cat. no. BD1000 S-250) and blocked in Background Sniper solution (Biocare, Cat. no. BS966 H). After washing, the samples were incubated with primary antibodies (Supplementary Table 2). After incubation, the samples were washed, incubated with horseradish peroxidase (HRP)-conjugated secondary antibodies, and then they were stained with hematoxylin and eosin (H&E) to provide the background.

### RT-qPCR analysis

RT-qPCR analysis was carried out according to a method described previously [11]. In brief, the total RNA obtained from each tissue was reverse transcribed using the QuantiTect RT kit (Qiagen, Cat. no. 205311). To assess the levels of gene expression, RT-qPCR was conducted using a real-time PCR system (Applied Biosystems ViiA 7) and SYBR Green as the double-stranded DNA-specific fluorescent dye (Bio-Rad, Cat. no. 1708880, Supplementary Table 2). Glyceraldehyde 3-phosphate dehydrogenase (GAPDH) was used as an internal control to normalize the RT-qPCR efficiency and quantify the gene expression levels. RT-qPCR was performed on each sample independently and in triplicate.

### Rearrangement analysis of TCR and BCR loci

DNA samples were extracted from the spleen, lymph node, and thymus of RAG-2 bKO and wild-type (WT) pigs. PCR analysis for rearrangements in the T-cell receptor (TCR)-β and immunoglobulin H (IgH) loci of B-cell receptor (BCR) was performed according to a previously reported method (Huang et al., 2014). Briefly, DNA samples were extracted from the thymus, spleen, and lymph node of the WT and *RAG-2* KO pigs. The primers for TCR and IgH are shown in Supplementary Table S2. The PCR conditions were as follows: 98°C for 5 min; 30 cycles of 98°C for 10 s, 68°C for 30 s, and 68°C for 1 min.

### RNA amplification, labeling, and hybridization to agilent/illumina microarrays

The samples for microarrays were prepared according to the manufacturer’s instructions. These experiments were performed by Macrogen (Seoul, Korea). In briefly, the porcine (V2) gene expression 4x44K microarray (Agilent Technologies, Cat. no. G2519F-026440) and Illumina MouseRef-8 v2 Expression BeadChip (Illumina) was used. Labeling was carried out using a low RNA fluorescent linear amplification kit (Agilent Technologies, Cat. no. 5184-3523). The sample and control RNAs were labeled with Cy-3 and Cy-5, respectively for pig and biotin for mouse. Fragmentation was carried out by incubation at 60°C for 30 min in a fragmentation buffer, and the process was stopped by the addition of an equal volume of hybridization buffer. Hybridization was carried out at 60°C for 17 h in a hybridization oven. The hybridized array was scanned using a SureScan microarray scanner (Agilent Technologies, Cat. no. G4900DA) and Illumina Bead Array Reader Confocal Scanner. The TIFF image generated was loaded into the Feature Extraction software (Agilent Technologies, Cat. no. G4460AA) for feature data extraction, the data analyses were performed using the GeneSpring software (Agilent Technologies, Cat. no. G3778AA) and Illumina GenomeStudion v2011.1 (Gene Expression Module V1.9.0).

### RNA-sequence analysis

The library was prepared using an Illumina TruSeq Stranded mRNA sample prep kit (Illumina) according to the manufacturer’s instructions. Quality and band size of the libraries were assessed using the Agilent 2100 Bioanalyzer (Agilent). Libraries were quantified using qPCR using CFX96 real-time system (Bio-Rad). After normalization, the prepared library was sequenced using the 75-bp-length paired-end (PE) reads using an Illumina NextSeq500 sequencer (Illumina Inc., San Diego, CA, USA). Potentially existing sequencing adapters and raw quality bases in the raw reads were trimmed using the Cutadapt software [14]. The option -a AGATCGGAAGAGCACACGTCTGAACTCCAGTCAC and -A AGATCGGAAGAGCGTCGTGTAGGG AAAGAGTGTAGATCTCGGTGGTCGCCGTATCATT were used for the common adapter sequence of the Illumina TruSeq adapters and the option -q 0, -m 20, and -O 3 was used for trimming low-quality 5′ and 3′ ends of the raw reads. To quantify the mapped reads on the human reference genome into the gene expression values, the Cufflinks software [20] with the strand-specific library option, —library-type = fr-firststrand and other default options was used. The gene annotation of the human reference genome hg19 from UCSC genome (https://genome.ucsc.edu) in GTF format was used as gene models, and the expression values were calculated in Fragments Per Kilobase of transcript per million fragments mapped (FPKM) unit.

### Pathway studio and GO analysis

Pathway studio analysis was performed as described previously [21] and performed using the Inslicogen. To identify molecular pathways, we arranged the data using the Pathway studio 9.0 software (Ariadne Genomics). GO analysis of the significant probe list was performed using PANTHER (http://www.pantherdb.org) using text files containing the Gene ID list and accession numbers of the Illumina probe ID. All data analyses and visualization of differentially expressed genes were conducted using R 2.4.1 (http://www.r-project.org). In addition, DAVID Functional Annotation Bioinformatics Microarray Analysis tools (http://david.abcc.ncifcrf.gov) were used to study the biological function of the regulated genes.

### Statistics

All experimental data are presented as the means ± standard deviation (SD). P-values were calculated using a one-way analysis of variance (ANOVA) and Fisher’s post-test. In all experiments, *P* < 0.05, *P* < 0.01, and *P* < 0.001 were considered significant.

### Data availability

All original microarray data of the *Rag2* KO mouse and the *RAG-2* bKO pig were deposited in the Gene Expression Omnibus (GEO GSE: 98102 for mouse; GEO GSE: 97505 for pig) of the National Center for Biotechnology Information (NCBI).

## SUPPLEMENTARY MATERIALS FIGURES AND TABLES




