# Supplementary material for: Recombination activating gene-2null severe combined immunodeficient pigs and mice engraft human induced pluripotent stem cells differently
Source: Oncotarget. 2017 Sep 2;8(41):69398–407. doi: 10.18632/oncotarget.20626 (PMC5642487; doi:10.18632/oncotarget.20626)
Supplement: Supplementary file 1 [file oncotarget-08-69398-s001.pdf]

Recombination activating gene-2^{null} severe combined immunodeficient pigs and mice engraft human induced pluripotent stem cells differently

SUPPLEMENTARY MATERIALS

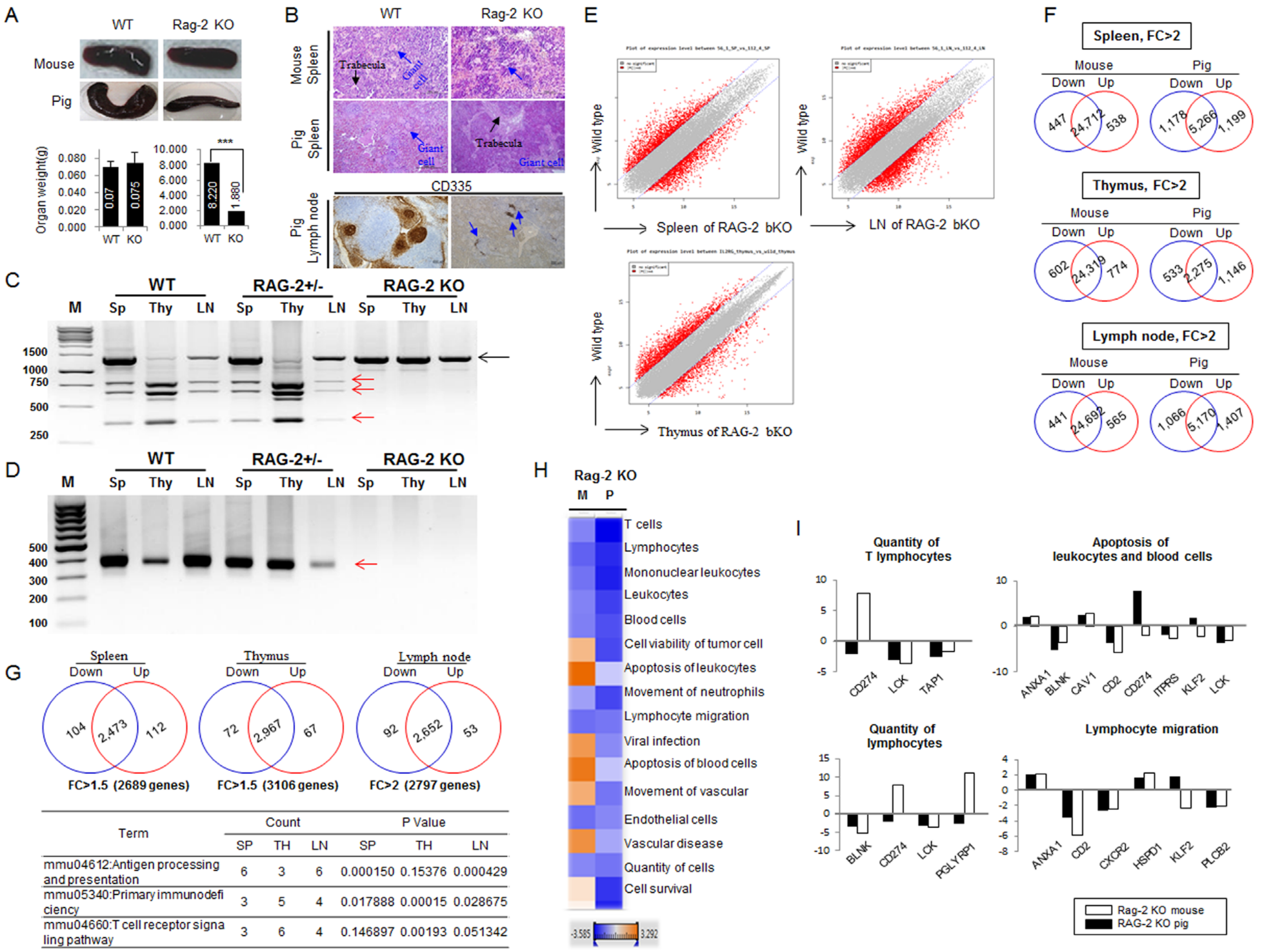

Supplementary Figure 1: (related to Figure 1), Histological and microarray gene expression analyses of recombination activating gene-2 (RAG-2) biallelic knockout (bKO) pigs, Rag-2 KO mice, and their wild-type (WT) counterparts. (**A**) Rag-2 KO mice showed similar spleen weight, whereas RAG-2 bKO pigs showed significantly reduced spleen weight, relative to WT animals. (**B**) Pathological analysis of spleens of Rag-2 KO mice and RAG-2 bKO pigs compared with their WT counterparts. Comparison of cluster of differentiation 335 (CD335) expression patterns in lymph nodes of WT and RAG-2 bKO pigs. Of note, natural killer (NK) cells were rarely detected in RAG-2 bKO pig. (**C**) Rearrangement of T-cell receptor-β (TCR-β) gene in spleen, thymus, and lymph node of RAG-2 bKO pigs. For V(D)J recombination analyses in the lymphoid organs, polymerase chain reaction (PCR) analysis was performed according to previously reported methods (3). PCR product of bKO pigs showed only single band (black arrow), whereas WT pigs showed additional bands (red arrows). M, Sp, Thy, and LN indicate size marker, spleen, thymus, and lymph node, respectively. (**D**) Rearrangement analysis of immunoglobulin H (IgH) gene in the spleen, thymus, and lymph node. PCR analysis was performed according to previous report (3). The rearranged band (arrow) was detected in the WT pigs, whereas lymphoid organs from RAG-2 bKO pigs did not show amplification of any product. M, Sp, Thy, and LN indicate size marker, spleen, thymus, and lymph node, respectively.

(**E**) Volcano plot of the differentially expressed mRNAs between WT and RAG-2 pig lymphoid organs**.** Spleen-, lymph node-, and thymus-derived microarray data of WT pigs was compared with those of RAG-2 bKO pigs. Red indicates high or low expression and gray indicates not altered expression. (**F**) Expression profiles of upregulated and downregulated genes in the spleen, thymus, and lymph node (fold change [FC] > 2) of RAG-2 bKO pigs and Rag-2 KO mice. Blue and red circles in Venn diagrams indicate downregulated and upregulated gene expression, respectively. (**G**) Expression profiles of upregulated and downregulated genes commonly expressed in both RAG-2 bKO pig and Rag-2 KO mouse lymphoid organs. Top three enriched biological processes, which were upregulated in Rag-2 KO mice but downregulated in RAG-2 bKO pigs, were categorized according to their functional role. (**H**) Heatmap showing strict segregation in KEGG pathway analysis of Rag-2 KO mice and RAG-2 bKO pigs. (**I**) Microarray expression data of Rag-2 KO mice and RAG-2 bKO pigs .

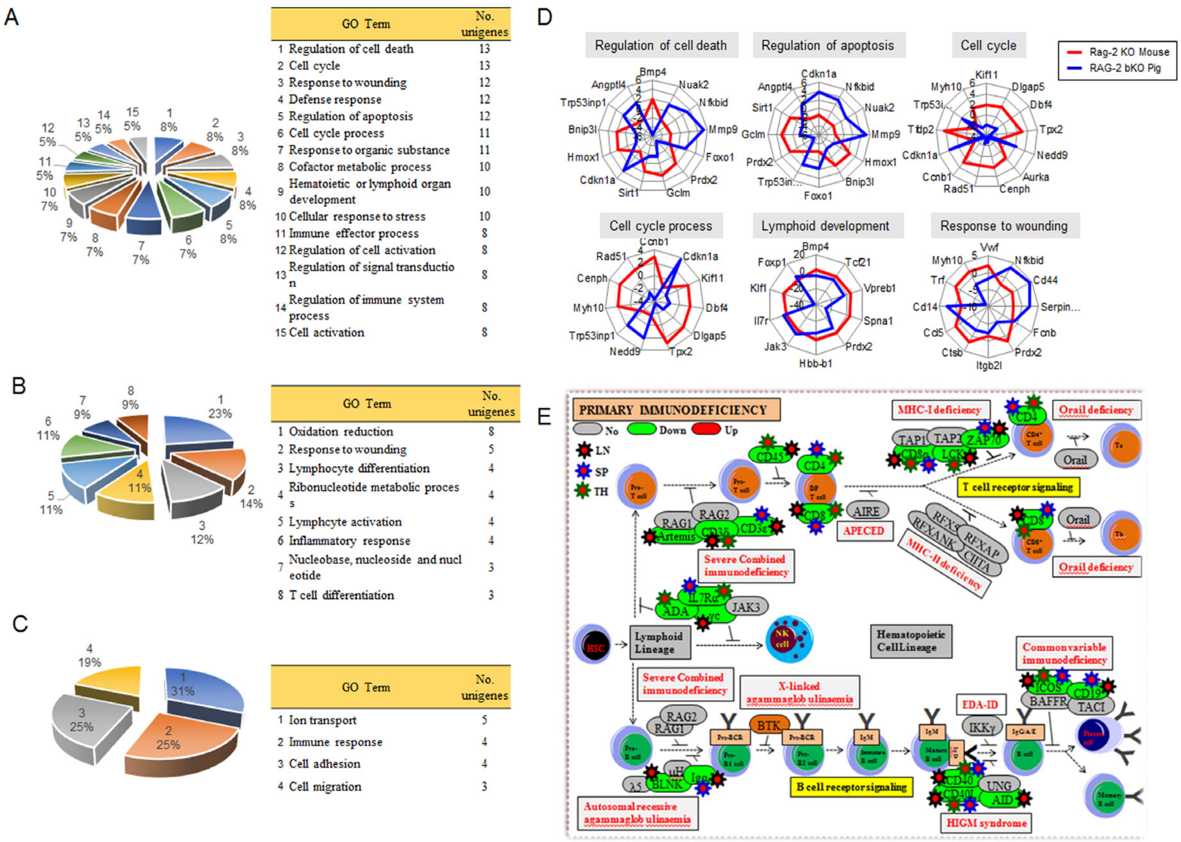

Supplementary Figure 2: (related to Figure 3), Differential gene expression profiles in recombination activating gene-2 (RAG-2) biallelic knockout (bKO) pig and Rag-2 KO mouse lymphoid organs. Expression profiles of upregulated and downregulated genes in Rag-2 KO mouse and RAG-2 bKO pig spleen (**A**), lymph node (**B**), and thymus (**C**). Data were analyzed using DAVID bioinformatics resources. Top enriched processes are presented in each table: cell death and cell cycle for spleen, oxidation-reduction, and response to wounding for lymph node, and ion transport and immune response for thymus. (**D**) Differentially expressed genes from each sub-gene ontology (GO) term presented as radar chart of spleen. Red and blue lines indicate Rag-2 KO mouse and RAG-2 bKO pig, respectively. (**E**) Primary immunodeficiency pathway analysis. Up/downregulated transcripts are depicted in red/green while gray color indicates not altered or detected gene expression.

For Supplementary Tables see in Supplementary Files
