# Supplementary material for: Recombination activating gene-2null severe combined immunodeficient pigs and mice engraft human induced pluripotent stem cells differently
Source: Oncotarget. 2017 Sep 2;8(41):69398–407. doi: 10.18632/oncotarget.20626 (PMC5642487; doi:10.18632/oncotarget.20626)
Supplement: Supplementary file 2 [file oncotarget-08-69398-s002.docx]

**Supplementary Table 1. Differentially expressed genes (recombination activating gene-2 [*RAG-2*] biallelic knockout [bKO] pig/*Rag-2* KO mouse values)**

| **GO: Id (Name)** | **DEG (*RAG-2* bKO pig / *Rag-2* KO mouse values)** |
| --- | --- |
| GO:0010941  (Regulation of cell death) | *BMP4* (-5.68/1.92), *NUAK2* (1.50/-1.95), *NFKBID* (3.08/-2.44), *MMP9* (5.13/-2.02), *FOXO1* (6.63/-2.11), *PRDX2* (-4.85/1.66), *GCLM* (-1.54/2.69), *SIRT1* (-1.62/1.68), *CDKN1A* (3.67/-1.54), *HMOX1* (-2.07/2.36), *BNIP3L* (-2.26/1.97), *TRP53INP1* (1.77/-1.51), *ANGPTL4* (1.89/-1.76) |
| GO:0007049  (Cell cycle) | *KIF11* (-1.91/1.88), *DLGAP5* (-2.10/2.18), *DBF4* (-1.55/1.78), *TPX2* (-3.60/2.87), *NEDD9* (2.11/-1.66), *AURKA* (-3.47/1.51), *CENPH* ( -2.87/2.22), *RAD51* (-2.46/1.88), *CCNB1* (-3.76/2.85), *CDKN1A* (3.67/-1.54), *TFDP2* (-3.34/4.34), *TRP53INP1* (1.77/-1.51), *MYH10* (-2.36/1.91) |
| GO:0009611  (Response to wounding) | *VWF* (-1.66/1.96), *NFKBID* (3.08/-2.44), *CD44* (4.31/-1.77), *SERPINA1B* (2.44/-2.53), *FCNB* (2.26/-1.63), *PRDX2* (-4.85/1.66), *ITGB2* (-2.11/1.66), *CTSB* (-2.05/1.86), *CCL5* (-1.62/1.72), *CD14* (2.57/-1.82), *TRF* (-8.72/1.54), *MYH10* (-2.36/1.91) |
| GO:0006952  (Defense response) | *NFKBID* (3.08/-2.44), *CD44* (4.31/-1.77), *SERPINA1B* (2.44/-2.53), *FCNB* (2.26/-1.63), *BNIP3L* (-2.26/1.97), *H2-AA* (-2.80/2.36), *MPO* (24.39/-1.79), *PRDX2* (-4.85/1.66), *ITGB2* (-2.11/1.66), *CCL5* (-1.62/1.72), *CD14* (2.57/-1.82), *TRF* (-8.72/1.54) |
| GO:0042981  (Regulation of apoptosis) | *CDKN1A* (3.67/-1.54), *NFKBID* (3.08/-2.44), *NUAK2* (1.50/-1.95), *MMP9* (5.13/-2.02), *HMOX1* (-2.07/2.36), *BNIP3L* (-2.26/1.97), *FOXO1* (6.63/-2.11), *TRP53INP1* (1.77/-1.51), *PRDX2* (-4.85/1.66), *GCLM* (-1.54/2.69), *SIRT1* (-1.62/1.68), *ANGPTL4* (1.89/-1.76) |
| GO:0022402 (Cell cycle process) | *CCNB1* (-3.76/2.85), *CDKN1A* (3.67/-1.54), *KIF11* (-1.91/1.88), *DBF4* (-1.55/1.78), *DLGAP5* (-2.10/2.18), *TPX2* (-3.60/2.87), *NEDD9* (2.11/-1.66), *TRP53INP1* (1.77/-1.51), *MYH10* (-2.36/1.91), *CENPH* (-2.87/2.22), *RAD51* (-2.46/1.88) |
| GO:0010033  (Response to organic substance) | *SERPINA1B* (2.44/-2.53), *SERPINA1D* (2.44/-1.78), *HMOX1* (-2.07/2.36), *FOXO1* (6.63/-2.11), *SERPINA3F* (1.88/-2.65), *NFKBIA* (4.54/-1.89), *PRDX2* (-4.85/1.66), *DEK* (-2.46/4.19), *CCL5* (-1.62/1.72), *CD14* (2.57/-1.82), *TRF* (-8.72/1.54) |
| GO:0051186  (Cofactor metabolic process) | *CES3* (1.72/-1.74), *HMOX1* (-2.07/2.36), *HMBS* (-9.42/2.22), *IDH2* (-1.95/3.31), *UROS* (-4.35/2.52), *GSTT1* (-2.22/1.57), *SPNA1* (-27.22/3.42), *IDH1* (2.42/1.51), *PPOX* (-5.44/2.52), *GCLM* (-1.54/2.69) |
| GO:0048534  (Hemopoietic or lymphoid organ development) | *BMP4* (-5.68/1.92), *TCF21* (-3.36/2.03), *VPREB1* (-3.99/5.05), *SPNA1* (-27.22/3.42), *PRDX2* (-4.85/1.66), *HBB-B1* (-5.78/2.23), *JAK3* (2.73/-2.17), *IL7R* (1078/-1.82), *KLF1* (-37.72/1.97), *FOXP1* (1.84/-3.39) |
| GO:0033554  (Cellular response to stress) | *FCER1A* (2.13/-1.64), *CDKN1A* (3.67/-1.54), *NUAK2* (1.50/-1.95), *HMOX1* (-2.07/2.36), *MPO* (24.39/-1.79), *PRDX2* (-4.85/1.66), *CAT* (-3.36/8.27), *SIRT1* (-1.62/1.68), *RAD51* (-2.46/1.88), *ANGPTL4* (1.89/-1.76) |
| GO:0002252  (Immune effector process) | *FCNB* (2.26/-1.63), *VPREB1* (-3.99/5.05), *BNIP3L* (-2.26/1.97), *H2-AA* (-2.80/2.36), *MPO* (24.39/-1.79), *PRDX2* (-4.85/1.66), *IL7R* (1078/-1.82), *FOXP1* (1.84/-3.39) |
| GO:0050865  (Regulation of cell activation) | *FCER1A* (2.13/-1.64), *CDKN1A* (3.67/-1.54), *NFKBID* (3.08/-2.44), *HMOX1* (-2.07/2.36), *CD274* (11.66/-2.11), *H2-AA* (-2.80/2.36), *PRDX2* (-4.85/1.66), *IL7R* (1078/-1.82) |
| GO:0009968  (Negative regulation of signal transduction) | *RGS10* (-3.40/2.17), *GPC3* (-3.21/1.91), *NFKBID* (3.08/-2.44), *SOCS3* (7.26/-1.63), *RGS4* (2.12/-1.62), *NFKBIA* (4.54/-1.89), *PRDX2* (-4.85/1.66), *ADRBK1* (1.81/-1.70) |
| GO:0002684  (Positive regulation of immune system process) | *FCER1A* (2.13/-1.64), *CDKN1A* (3.67/-1.54), *FCNB* (2.26/-1.63), *KLRK1* (-3.68/1.58), *H2-AA* (-2.80/2.36), *NFKBIA* (4.54/-1.89), *IL7R* (1078/-1.82), *FOXP1* (1.84/-3.39) |
| GO:0001775  (Cell activation) | *VWF* (-1.66/1.96), *SBNO2* (2.19/-1.51), *VPREB1* (-3.99/5.05), *KLRK1* (-3.68/1.58), *PRDX2* (-4.85/1.66), *ITGB2* (-2.11/1.66), *IL7R* (1078/-1.82), *FOXP1* (1.84/-3.39) |

**Supplementary Table 2. List of antibodies, primer sets, reagents, and software used**

| **Antibodies** | **Company** | **Catalog numbers** |
| --- | --- | --- |
| CD3 | DAKO (1/400) | IS50330-2 |
| CD4 | LSbio (1/100) | LS-C116954 |
| CD8 | LSbio (1/100) | LS-c196471 |
| CD20 | Leica (prediluted) | PA0906 |
| CD45R/B220 | Leica (prediluted) | PA0146 |
| GFAP | Leica (prediluted) | PA0026 |
| β-catenin | Leica (prediluted) | PA0083 |
| Desmin | Leica (prediluted) | PA0032 |
| CD34 | Leica (prediluted) | PA0212 |
| CD45 | Leica (prediluted) | PA0042 |
| CD8 | Leica (prediluted) | PA0183 |
| CD20 | Leica (prediluted) | PA0359 |
| NKp46 | BIOSS (1/100) | BS-10027R |
| OCT3/4 | Santacruz (1/200) | SC-9081 |
| vWF | DAKO (1/100) | IS52730-2 |
| NSE | Milipore (1/200) | AB951 |
| SMA | Abcam (1/200) | AB7817 |
| SSEA3 | Milipore (1/200) | MAB4303 |
| SOX2 | Abcam (1/200) | Ab97959 |
| **Deposited Data** | **Publications** | **NCBI GEO numbers** |
| Rag2 KO mouse | This paper | GSE98102 |
| RAG2 KO pig | This paper | GSE97505 |
| **Animals** | **Company** | **Catalog numbers** |
| *Rag2* KO mouse  (B6.129S6-*Rag2^tm1Fwa^* N12) | Taconic Farms Inc. | RAGN12 |
| *Rfp/Rag-2* KO mouse  (B6.129S6-*Rag2^tm1Fwa^Prf1^tm1Clrk^* N12) | Taconic Farms Inc. | 1177 |
| *RAG2* KO pig | House made | N/A |
| **Software and algorithms** | **Address** | **version** |
| Studio pathway | <https://www.elsevier.com> | - |
| PANTHER | <http://pantherdb.org/> | - |
| R software | https://www.r-project.org/ | 2.4.1 |
| DAVID | https://david.ncifcrf.gov/ | 6.7 |
| GeneSpring software | GeneSpring software | G3778AA |
| **Main Reagents** | **Company** | **Catalog numbers** |
| Matrigel solution | BD | Cat. no. 354277 |
| mTeSR1 medium | StemCell | Cat. no. 85821, 85852 |
| Borg Decloaker | Biocare | Cat. no. BD1000 S-250 |
| Background Sniper solution | Biocare | Cat. no. BS966 H |
| EnVisionTM+ system | Dako | Cat. no. K4002 |
| K4002 | Biocare | Cat. no. RAEC810 |
| RNeasy Mini Kits | Qiagen | Cat. no. 74104 |
| QuantiTect Reverse Transcription Kit | Qiagen | Cat. no. 205311 |
| SYBR Green | Bio-Rad | Cat. no. 1708880 |
| **Genes** | **Sequences** | **Species** |
| *hOct3/4* | F-GAGAAGGAGAAGCTGGAGCA  R-AATAGAACCCCCAGGGTGAG | Human |
| *hSSEA3* | F-CCAAGAGCGTCCCATACATT  R-GCCTGCCAGTAGTCCAAGAG | Human |
| *hSOX2* | F-AACCCCAAGATGCACAACTC  R-CGGGGCCGGTATTTATAATC | Human |
| *hCD34* | F-CACCCTGTGTCTCAACATGG  R-GGCTTCAAGGTTGTCTCTGG | Human |
| *hCD45* | F-TGCAAACATCACTGTGGATT  R-GGAAACAGACGCATTTTTACA | Human |
| *hMFN1* | F-GCTGGCTAAGAAGGCGATTA  R-TCCCCTTCTGGAGGTTAGAAA | Human |
| *hGAPDH* | F-CATGTACGTTGCTATCCAGGC  R-CTCCTTAATGTCACGCACGAT | Human |
| *dNKp30* | F-ATCCTGTGCTCTCTGGGTGT  R-GTCCCATTCCTCACCTCCTT | Degenerative primers |
| *dGATA3* | F-ACAGACCCCTGACCATGAAG  R-GGAGATGTGGCTGAGAGAGG | Degenerative primers |
| *dIRF2* | F-GAAAGCATCAACCGGGAGTA  R-GCTCAGATAAGGGCAGCATC | Degenerative primers |
| *dETS1* | F-CCTCCTACGACAGCTTCGAC  R-AGAAACTGCCACAGCTGGAT | Degenerative primers |
| *pTCR* | F-GCTGCTCTGGTGGTTTCTCAC  R-CACCAGTGCCCAAGTCTTAGC | Pig |
| *pIgH* | F-GAGGAGAAGCTGGTGGAGT  R-TGAGGACACGACGACTTCAA | Pig |
| Inner Control for IgH | F-CTGAGAACTCACGTCCAGTGC  R-CTGGCCCTAGACCTTTAGACC | Pig |
| *pNKp46* | F-GTGAAGCTCCTGGTCAAAGG  R-CTCTTCCTGTGGAGGCAGTC | Pig |
| *pNKG2A* | F-TCATTACTTTTTGCTTGGCTTTT  R-CCGTCTCTCCTCAGTCCTTG | Pig |
| *pMEF1* | F-GGAGGAGCACTGTCTGAAGG  R-GATGTACTTGGGGCAGGTGT | Pig |
| *pEOMOS* | F-ACTGGTTCCCACTGGATGAG  R-GCAATGAACTGCGTTTCTGA | Pig |
| *pID2* | F-CCAGTGAGGTCCGTTAGGAA  R-GACAATAGTGGGGTGCGAGT | Pig |
| *pMCM4* | F-CGACGAGTTCGACAAGATGA  R-GGCAGCTGGATGTTTTCAAT | Pig |
| *pTBX21* | F-GGACCCAACTGTCAATTGCT  R-TCATGCTGACAGCTCGAAAT | Pig |
| *pCD57* | F-TTGCAATAGACATGGCTGGA  R-GTCRGTGAAGCCCTTCTTSC | Pig |
| *pETS1* | F-CCTCCTACGACAGCTTCGAC  R-AGAAACTGCCACAGCTGGAT | Pig |
| *pCX3CR1* | F-TGGATGAGGCTCAGACACAG  R-CCATCCCAAAGGAAGTCTGA | Pig |
| *pActin* | F-GCACCTCAACCCGCTCCTAG  R-GAGGGCAGTAGCATCGCTTTA  G TG | Pig |
| *mNKp46* | F-GAGAGACCAAAGCCATCGAG  R-TTGTGGCAGTCTTCAGTTGG | Mouse |
| *mNKG2A* | F-AACCAGCACAGGAAACCAAG  R-CCAGGGTACCAGCAATGAGT | Mouse |
| *mMEF1* | F-GCCAATTCCCATCTGAAGAA  R-GTCGCTCAAACTCCTCCTTG | Mouse |
| *mEOMOS* | F-TTCCGGGACAACTACGATTC  R-CTCTGTTGGGGTGAGAGGAG | Mouse |
| *mID2* | F-CTCCAAGCTCAAGGAACTGG  R-ATTCAGATGCCTGCAAGGAC | Mouse |
| *mMCM4* | F-GACCCTCGTACTGGCATTGT  R-GTGTCAGACTGTCCCCGAAT | Mouse |
| *mTBX21* | F-CCTGGACCCAACTGTCAACT  R-AACTGTGTTCCCGAGGTGTC | Mouse |
| *mCD57* | F-TCCTTCTCCGTGTTGGACAT  R-GAGTCCCCAGCACTTGTAGG | Mouse |
| *mCX3CR1* | F-CACCATTAGTCTGGGCGTCT  R-GATGCGGAAGTAGCAAAAGC | Mouse |
| *mGAPDH* | F-AGGTCGGTGTGAACGGATTTG  R-TGTAGACCATGTAGTTGAGGTCA | Mouse |
